# Giant mediastinal mature teratoma with increased exocrine pancreatic activity presenting in a young woman: a case report

**DOI:** 10.1186/1752-1947-5-238

**Published:** 2011-06-27

**Authors:** Franco Stella, Fabio Davoli

**Affiliations:** 1 Department of Thoracic Surgery, "S. Orsola-Malpighi" Hospital, University of Bologna, Bologna, Italy

## Abstract

**Introduction:**

Mediastinal mature teratoma is a benign, slow-growing tumor typically affecting 20- to 40-year-old adults. Fluid examination from the cystic masses rarely shows enzymatic activity as we describe in this report.

**Case presentation:**

We report a case of a giant mediastinal germ cell tumor (measuring 15 cm × 14 cm × 8 cm) detected in a 35-year-old Caucasian woman. Microscopic examination showed that the lesion resembled a mature cystic teratoma with areas of pancreatic tissue with mature ductal and acinar structures intermixed with islets of Langerhans. Fluid from the cysts in the mass was examined after removal showed amylase activity of 599 U/l despite normal serum levels. The post-operative period was free of complications, and the patient was discharged on post-operative day 10.

**Conclusion:**

Complete surgical removal is the treatment of choice for mature cystic teratomas, with optimal results and acceptable surgical risk. Exocrine pancreatic function may be an aid to pre-operative or intra-operative diagnosis; however, these findings have no impact on survival or the therapeutic pathway.

## Introduction

Primary germ cell tumors (GCTs) of the mediastinum are rare tumors (10% to 15% of all mediastinal tumors), and they usually appear during the third to fourth decade of life [1]. GCTs are predominantly found in gonads, and the anterior mediastinum is the common extragonadal site [2]. The majority of mediastinal teratomas are mature teratomas that are histologically well-defined and benign [3]. Malignant GCTs account for less than 1% of all mediastinal tumors, and mature teratomas account for approximately 8% [1].

## Case report

**Figure 1:**
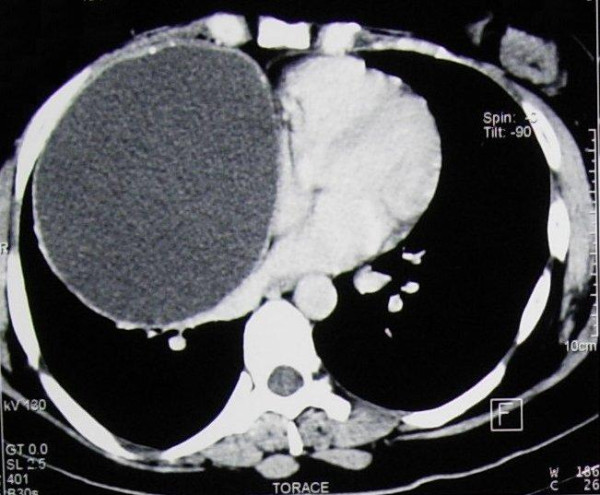
**Chest computed tomographic scan revealing the mass compressing the mediastinal vessels**.

We present a case of a giant mature teratoma of the anterior mediastinum in a 35-year-old Caucasian woman. Following the onset of an episode of influenza associated with productive cough, the patient, an ex-smoker, underwent a chest X-ray which revealed the presence of a severe increase of the frontal cardiac area, with particular involvement of the atrial sectors and evident divarication of the sternal angle. Subsequent transthoracic echocardiography showed a rounded parenchyematous extra-cardial formation measuring approximately 9 cm in diameter and pressing against the lower posterior wall of the right atrium, with the chambers and valves of the heart being within the norm (Ejection Fraction = 71%), and an anomalous movement of the inter-ventricular septum. A thoracic computed tomographic scan with contrast medium confirmed the presence of a voluminous expansive formation with a maximum diameter of 125 mm, liquid contents, clear contours, multiple compartments, and parietal calcifications. This tumor was causing compression of the adjacent pulmonary parenchyma, the vascular and bronchial structures of the right lung (subsequently confirmed with fibrobronchoscopy), and the superior vena cava, without signs of infiltration (Figure 1). The tumor markers tested while the patient was hospitalized (α-fetoprotein, β-HCG, and CA 19-9) were within normal ranges. Her respiratory function tests showed a prevalent, obstructive type, slight ventilatory incapacity: forced expiratory volume in one second was 2.63 (73% of the former value), forced vital capacity was 3.63 (87% of the former value), and blood gases at baseline and after stress were within the normal range. The day after the patient was admitted to our hospital, she underwent surgery to excise the mediastinal mass with a median sternotomy approach.

Once the mediastinal cavity was opened, it was possible to see the presence of the voluminous tumor, which occupied most of the right hemithorax with compression of the adjacent pulmonary parenchyma. There were also widespread adhesions to the superior vena cava, the ascending aorta, the pericardium, and part of the lower pulmonary lobe, where an atypical resection of pulmonary parenchyma was performed.

The lesion measured 15 cm × 14 cm × 8 cm and showed smooth, regular external contours. The cut surface of the mass was cystic, filled with a yellowish white proteinaceous material admixed with hair. A thickened area of the cyst wall measuring 6.5 cm across was composed of a more solid white fibrous tissue, which also contained multiple small cysts filled with yellowish material. Fluid from the cysts in the mass was examined after removal, showing an amylase activity of 599 U/l despite serum levels being normal.

**Figure 2:**
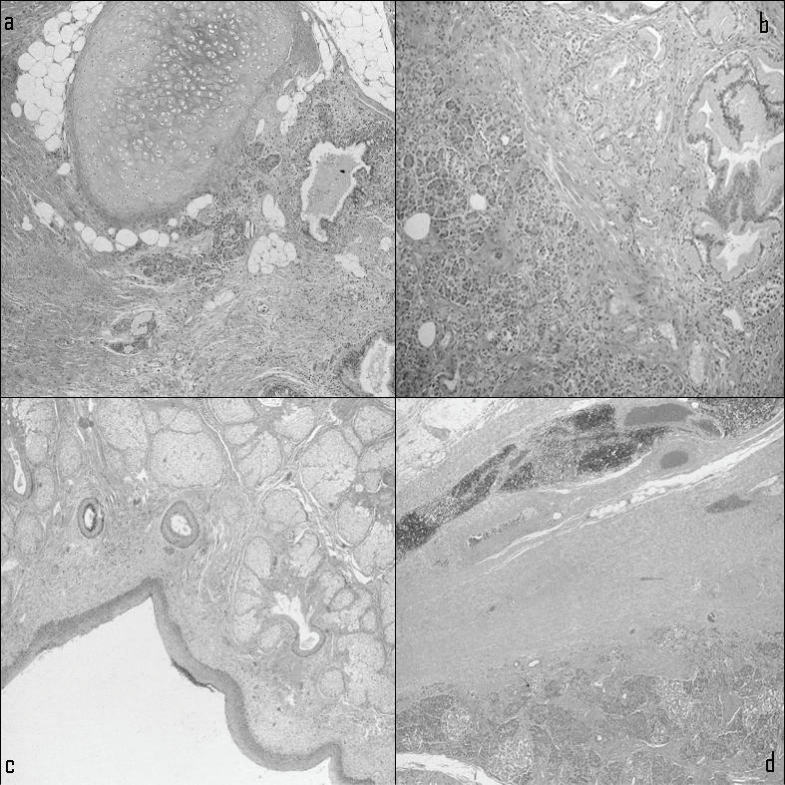
**Microscopic histopathological examination showing various tissue components of the mature cystic teratoma of the mediastinum**. **(a) **Cartilaginous and adipose tissue admixed with smooth muscle fibers and lined by squamous and respiratory epithelium. **(b) **Pancreatic tissue. **(c) **Skin appendages. **(d) **Periphery of the lesion surrounded by residual thymic tissue with rare Hassall's corpuscles.

Microscopically, the lesion resembled a mature cystic teratoma, which is more commonly seen in the ovary. The cystic wall was predominantly lined by a squamous epithelium associated with sebaceous glands, hair follicles, and a chronic inflammatory reaction (Figure 2). Other areas of the cyst showed a monostratified ciliated epithelium, cartilage, and mucinous glands recapitulating respiratory tract structures. More solid areas of the mass revealed pancreatic tissue with mature ductal and acinar structures intermixed with islets of Langerhans. In addition, in adipose tissue at the periphery of the mass, a thymic residue with prominent Hassall's corpuscles was identified. Careful examination of the mass did not reveal the presence of immature tissue. The post-operative period was uneventful, and the patient was discharged on post-operative day 10.

## Conclusion

Mature teratomas typically occur in young patients (mean age, 27 years), with approximately equal frequency in men and women (> 90% of malignant germ cell tumors occur in men). Histologic examination of mature teratomas reveals malignant transformation in less than 1% of cases and is usually characterized by the malignant degeneration of the squamous epithelium [1]. Although the actual mechanism of development is unclear, it is believed that these lesions consist of primordial germ cells which stray into midline extragonadal areas in the migration during embryonic development [4]. Most patients with germ cell tumors in the mediastinum are asymptomatic, so these neoplasms are usually discovered by accident during routine chest X-ray examinations [3]. Patients may present with chest, back, or shoulder pain; dyspnea; fever; pleural effusion; cough; and bulging of the chest wall. Less commonly, tumors become infected or may rupture into adjacent organs, such as the lung, bronchial tree, or pleural and pericardial space [2,5]. Symptoms can also derive from the pressure exerted on the surrounding tissue (vena cava syndrome), and occasionally fluid examination from the cystic mass shows physiological activity. Some authors [6,7] have suggested that exocrine secretion by pancreatic tissue and leakage of digestive enzymes from intestinal or salivary tissue are due to non-infective inflammation around the mass. Others [8] believe that exocrine pancreatic function may be an aid to pre-operative or intra-operative diagnosis. Anyway, these findings have not had an impact on survival or on the therapeutic pathway. For cases of pure mature cystic teratomas, complete surgical removal alone is the treatment of choice, with optimal results and acceptable operative risk.

## Consent

Written informed consent was obtained from the patient for publication of this case report and any accompanying images. A copy of the written consent is available for review by the Editor-in-Chief of this journal.

## Competing interests

The authors declare that they have no competing interests.

## Authors' contributions

FS chose the surgical strategy (median sternotomy) and played the greatest role during the surgical operation.

FD was a major contributor to the writing of the manuscript and to collecting all the data about the patient that suggested the particularly high levels of amylase in the cystic fluid; he also reviewed the literature. All authors read and approved the final manuscript.
